# Factors Associated with Utilization of HIV Testing Services among Adolescents Aged 10-19 Years in Lira District, Northern Uganda: A Cross-Sectional Study

**DOI:** 10.1155/2021/9568148

**Published:** 2021-08-11

**Authors:** Deo Benyumiza, Joan Fidelia Amongin, Isaac Ochaba, Morish Adupa, Naume Abuch, Constance Babirye Banula, Samson Udho

**Affiliations:** Faculty of Health Sciences, Lira University, P.O. Box 1035, Lira, Uganda

## Abstract

**Background:**

HIV testing remains a problem among adolescents in low- and middle-income countries, and yet, HIV testing is a cornerstone in the fight against HIV. However, there is scanty literature on the utilization of HIV testing services by adolescents especially in rural settings. This study is aimed at determining the uptake of HIV testing services and associated factors among adolescents aged 10-19 years in Lira District, Northern Uganda.

**Methods:**

This was a cross-sectional study done among 277 randomly selected adolescents aged 10-19 years attending outpatient clinics in Pentecostal Assembly of God (PAG) Mission Hospital, Ngetta Health Center III, and Boroboro Health Center III. Data were collected using an interviewer-administered structured questionnaire. Data collected included sociodemographic characteristics, history of test and receipt of HIV results in the last three months, and facility-related factors affecting uptake of HIV testing services. Data analysis consisted of descriptive statistics, cross-tabulations, and logistic regression at a 95% level of significance in SPSS version 25.

**Results:**

The uptake of HIV testing services was 43% (119/277) among the study participants. Adolescents who had completed primary education (aOR: 5.47; 95% CI: 1.07-28.15; *p* = 0.042), are employed (aOR: 2.77; 95% CI: 1.16-6.60; *p* = 0.022), had used a condom in the last sexual intercourse (aOR: 4.46; 95% CI: 1.78-11.15; *p* = 0.001), and are involved in HIV testing outreaches (cOR: 10.86; 95% CI: 3.81-30.93; *p* ≤ 0.001) were more likely to uptake HIV testing services compared to those who had tertiary education, are unemployed, had never used a condom, and are not involved in HIV testing outreaches.

**Conclusion:**

Utilization of HIV testing services by adolescents aged 10-19 in Lira District, Northern Uganda, is generally low. The Ministry of Health should strengthen HIV testing services targeting adolescents to increase uptake of HIV testing services.

## 1. Introduction

HIV/AIDS remains a pandemic of public health interest [1]. Globally, it is estimated that 1.7 million adolescents between ages 10 and 19 are living with HIV with 170,000 new infections and 34,000 AIDS-related deaths [2]. Sub-Saharan Africa accounts for 73% of new HIV infections among adolescents occurring globally [2]. In Uganda, the prevalence of HIV among adolescents is still high at 3.3%, and yet, adolescents are key to achieving HIV targets in Uganda [3]. HIV continues to disproportionately affect the adolescent at a global, regional, and national level [2]. Adolescents are more vulnerable to HIV infection because of their risky sex behaviors, inexperience, and invincible tendency [4, 5].

HIV testing is a window for all HIV-related care and treatment services and an essential step in achieving “the UNAIDS 90-90-90 targets” among all age groups [5]. However, globally, only 35% of young people were aware of their HIV status in 2015 [6]. In sub-Saharan Africa, only 13% of female and 9% of male adolescents tested for HIV and received their results in the last 12 months [7]. In Uganda, only 39.4% of older adolescents (15-19 years) had tested and received their HIV results in the past 12 months [8]. This has prompted the government of Uganda through the Ministry of Health to adopt both community-based and facility-based HIV testing to scale up HIV testing [9].

However, the uptake of HIV testing in health facilities, including faith-based health facilities, has remained generally lower than that in community-based approaches [10]. This has been attributed to the attitude of healthcare workers, fear of stigmatization, and fear over HIV confidentiality [11]. Additionally, institutional grounding towards offering comprehensive HIV testing services (HTS) to young people also constitutes a major barrier to the provision of HTS although this is not well documented [12]. More efforts are still needed in terms of repositioning health facilities to accommodate all persons regardless of their occupation, sexual orientation, and age category. Additionally, factors such as individual and institutional biases that hinder adolescents from accessing health facilities need to be studied and addressed [12, 13].

There is a dearth of literature on the uptake of HIV testing services by adolescents in faith-based health facilities across Uganda. Previous studies on HIV testing among adolescents were largely carried out in either community or public health facility settings [4, 5, 14]. Faith-based health facilities particularly those founded on Christian beliefs are unique because of their religious beliefs which tend to disapprove of premarital sex and uphold abstinence. Such beliefs may impact the provision of HIV testing services to adolescents who are usually still unmarried. Lira District has a host of faith-based health facilities with the capacity to reach out to adolescents, and yet, little is known about the uptake of HIV testing services by adolescents in these facilities. The purpose of this cross-sectional study is to assess the utilization of HIV testing by adolescents and associated factors in faith-based health facilities in Lira District, Northern Uganda.

## 2. Materials and Methods

### 2.1. Study Design and Setting

A cross-sectional study with a quantitative method of data collection was conducted in April 2021. The study was conducted in outpatient clinics of three faith-based health facilities of Pentecostal Assemblies of God (PAG) Mission Hospital, Ngetta Health Center III, and Boroboro Health Center III. These facilities are located in Lira City, Northern Uganda, and they serve people both within Lira City and Lira District. All these facilities offer medical, surgical, pediatric, and obstetric services, and they have inbuilt outpatient clinics for each department including the HIV clinic. These facilities were chosen because of the high patient load and their proximity to institutions particularly schools dealing with adolescents who were likely to seek care from those facilities. Additionally, we hypothesized that faith-based health facilities could form barriers to the utilization of HIV testing services because of their religious beliefs on premarital sex, fidelity, and purity.

### 2.2. Study Population

This study was conducted among adolescents aged 10-19 years seeking non-HIV-related care in outpatient clinics of Pentecostal Assemblies of God (PAG) Mission Hospital, Ngetta Health Center III, and Boroboro Health Center III. Adolescents who were very ill, were in a hurry, or had come for minor surgical procedures were excluded from the study. This study focused on adolescents aged 10-19 years because of the negative attitudes, labeling, and judgmental behaviors of healthcare providers towards adolescents especially in faith-based facilities which may undermine utilization of HIV testing services.

### 2.3. Sample Size Estimation

Based on the rarity of adolescents in outpatient clinics in the selected health facilities, the sample size for this study was calculated using the Reid and Boore [15] formula for a finite population. The *z*-score (*z*) was 1.96, the margin of error (*e*) was 0.05, the prevalence (*p*) for the unknown population was 0.5, and the known population of outpatient adolescents (*N*) was 680. The initial calculated sample size was 384. This was reduced to 271 plus a 10% allowance for nonresponse.

### 2.4. Data Collection Method and Tools

We used interviewer-administered questionnaires to collect data with the help of three trained research assistants based at each of the three health facilities. The questionnaire was developed from a literature review of previous studies [4, 5, 14]. The questionnaire was reviewed and adjusted accordingly before data collection. The questionnaire collected information on the following: demographic characteristics of participants (age, sex, marital status, level of education, employment status, presence of a partner, number of partners, and age difference with primary partners), individual characteristics (being sexually active, use of condoms, and knowledge of partner's HIV status), facility-related factors (means of transport to a health facility, estimated distance to facility, opening time of facility, availability of service providers, waiting time, adequacy of HIV testing and counseling services, and facility involvement in HIV testing and counseling outreaches), and uptake of HIV testing and counseling services. The outcome variable for this study was self-reported HIV testing and receipt of test results within the past three months.

### 2.5. Data Collection Procedure

The study participants were selected using a consecutive sampling technique. During consecutive sampling, every subject meeting the inclusion criteria is selected until the required sample size is achieved. This sampling technique was chosen because of the limited number of adolescents in the outpatient clinics in the three facilities. Administration of the questionnaire with selected participants lasted between 25 and 40 minutes.

### 2.6. Data Management and Analysis

Every questionnaire was checked for completeness at the end of each interview. A data entry screen was created in SPSS version 23 with checks to ensure accuracy during entry. Data were scanned for out-of-range and missing values before commencing data analysis. Categorical variables were analyzed using descriptive statistics, cross-tabulation, and logistic regression at a 95% level of confidence. Variables with a *p* value < 0.2 at the binary logistic regression level were included in the multivariate logistic regression model. The model fitness was checked using the Hosmer-Lemeshow test at *p* > 0.05. The backward conditional logistic regression method was used to determine variables that were independently associated with the outcome variable.

### 2.7. Ethical Considerations

The research protocol was reviewed and approved by the Gulu University Research and Ethics Committee (GU-REC-2021-037) and Uganda National Council for Science and Technology (RESCLEAAR/01). Administrative clearance was also obtained from the office of the District Health Officer, Lira District, and facility administrations. Written informed consent was obtained from participants aged 18-19 years, while assent was sought from adolescents below 18 years of age. Privacy and confidentiality were maintained throughout the study by conducting the interviews in a private place, the use of codes instead of participants' names, and password protection of entered data.

## 3. Results

Out of the 271 prospective participants approached, 264 accepted to participate in the study giving a response rate of 97.4%. Additional participants were recruited to bring the actual sample size used in the study to 277.

### 3.1. Demographic and Individual Characteristics of Adolescents Aged 10-19 Years in Lira District, Northern Uganda

The median age of the participants was 17 years, and the interquartile range was 16 to 19 years of age. Out of the 277 participants interviewed, the majority were females (71%), were single (59%), had sexual partners (57%), and had never used a condom (58%), while more than three-quarters (91.1%) were sexually active (Table 1). Three-quarters of the participants (66%) had no formal education or have only completed primary level of education.

### 3.2. Uptake of HIV Testing Services among Adolescents Aged 10-19 Years in Lira District, Northern Uganda

Out of the 277 study participants interviewed, 43% (119/277) had tested for HIV within the last three months. Out of the 119 adolescents who had tested for HIV, utilization of HIV testing was more among females 85 (71.4%); single adolescents, 58 (48.7%); adolescents aged 18-19, 69 (58%); and those who had attained an ordinary level of education, 41 (34.5%).

### 3.3. Facility-Related Factors Affecting Utilization of HIV Testing Services among Participants

Almost three-quarters of the respondents (73%) reported that they had never been involved in any outreach to HIV testing and counseling organized by these services. More than half of the participants (53.4%) walked on foot to the health facilities to seek care (Table 2).

### 3.4. Factors Associated with HIV Testing among Adolescents Aged 10-19 Years in Lira District, Northern Uganda

In binary logistic regression, the factors that were statistically associated with HIV testing at *p* value < 0.05 were the age of participants (*p* = 0.006), marital status (*p* = 0.002), level of education (*p* = 0.006), employment status (*p* ≤ 0.001), having a partner (*p* ≤ 0.001), using a condom in the last sexual intercourse (*p* ≤ 0.001), knowledge of partner's HIV status (*p* ≤ 0.001), facility being opened by the time of arrival (*p* = 0.030), availability of service providers by time of arrival (*p* = 0.001), adequacy of HIV testing services at the facility (*p* ≤ 0.001), and involvement in HIV testing and counseling outreaches (*p* ≤ 0.001). Factors that were not statistically associated with HIV testing among study participants were sex of participants, age difference with primary partners, number of sexual partners, awareness about HIV testing services, means to health facilities, estimated and perceived distance to the facility, and waiting time.

In the multivariate logistic regression model, the factors that were independently associated with HIV testing among adolescents aged 10-19 years were completion of primary education (aOR: 5.47; 95% CI: 1.07-28.15; *p* = 0.042), being employed (aOR: 2.77; 95% CI: 1.16-6.60; *p* = 0.022), use of condom in the last sexual intercourse (aOR: 4.46; 95% CI: 1.78-11.15; *p* = 0.001), and involvement in HIV testing outreaches (cOR: 10.86; 95% CI: 3.81-30.93; *p* ≤ 0.001) (Table 3).

## 4. Discussion

In our study, the majority of the participants were females (71%) and older adolescents 18-19 years (49%). Uptake of HIV testing services by adolescents aged 10-19 years was relatively low at 43%. Adolescents who had completed primary education (aOR: 5.47; 95% CI: 1.07-28.15; *p* = 0.042), are employed (aOR: 2.77; 95% CI: 1.16-6.60; *p* = 0.022), had used a condom in the last sexual intercourse (aOR: 4.46; 95% CI: 1.78-11.15; *p* = 0.001), and are involved in HIV testing outreaches (cOR: 10.86; 95% CI: 3.81-30.93; *p* ≤ 0.001) were more likely to uptake HIV testing services compared to those who had tertiary education, are unemployed, had never used a condom, and are not involved in HIV testing outreaches. These findings suggest gaps in access to HIV testing services by adolescents in faith-based health facilities attributable to demographic, individual, and facility factors.

Consistent with previous studies conducted in Uganda [5] and Ethiopia [16], the majority of the participants in our study who tested for HIV were females and older adolescents. According to a systematic review and meta-analysis by Sharma et al. [17], gender differences exist in HIV testing, with men testing less frequently than women do. These observed differences could be attributed to multiple factors, including variations in the use of healthcare services, culturally defined masculinity, and the financial consequences that HIV poses to women and children [18]. Meanwhile, older adolescents are more autonomous and as such can make their own decisions including coming for HIV testing. These findings highlight the need to strengthen the mobilization of young and male adolescents to seek HIV testing services.

The 43% uptake of HIV testing services by adolescents from these facilities was higher than the 39% uptake reported in the Uganda Demographic and Health Survey report [8]. The high uptake of HIV testing services in this study could be explained by the fact that the majority of our participants had sexual partners and were aware of HIV testing services offered by these facilities. Results of our study were similar to those reported in Ethiopia [19] and Kenya [20] where the uptake of HIV testing services was 52% and 55%, respectively. However, the uptake of HIV testing among adolescents was dissimilar to that reported in other parts of Uganda [5] and Nigeria [21] where the uptake of HIV testing was significantly higher and lower at 82% and 14%, respectively. Disparities in findings could be because of the difference in duration within which the HIV test was ever taken. For example, the study conducted among 1439 adolescents aged 10–19 years attending nine public health facilities in five of the seven districts of Karamoja, Uganda, did not have a timeframe for self-report of HIV testing which could have contributed to the high prevalence of HIV testing. Based on the results of our study, more effort is still required by the government to meet the UNAIDS' first target of having 95% of people knowing their HIV serostatus by 2030.

One of the interesting findings of our study was that adolescents who had used a condom in their last sexual intercourse were eight times more likely to test for HIV compared to their counterparts who did not use a condom in the last sexual encounter. Condom use is a good proxy of knowledge about the prevention of HIV infection methods. Therefore, it is plausible that adolescents who used a condom during the last sexual intercourse are more likely to embrace other HIV prevention measures such as HIV testing before any penetrative sexual contact with a new partner. Nonetheless, studies conducted in Massachusetts, USA, and Soweto, South Africa, did not find any association between condom use and HIV testing [22, 23]. This may suggest a slightly different approach and overall dynamism regarding condom education and use across age groups. For instance, condom use may not seem appealing or relevant in some cases to those in stable relationships, particularly older age groups and those that live with their sexual partners as was the case in the South African study [22].

Ironically, adolescents who had completed primary level of education were five times more likely to test for HIV within the past three months compared to their other counterparts who had tertiary level education. Results of this study are inconsistent with a plethora of results of other studies across different contexts where higher levels of education were associated with HIV testing compared to a lower level of education [4, 5, 24, 25]. This misnomer could be explained by the fact that there is more emphasis on HIV/AIDS education and prevention in primary schools through a formal curriculum, use of drama, and signage displayed in school compounds that emphasize HIV/AIDS risks, prevention, and control. These strategies may lead to sustained high-risk perceptions among these adolescents whose knowledge base and risk perceptions may not be diluted through peer-peer education that happens in tertiary institutions. This finding suggests the need for continued HIV/AIDS education among adolescents in tertiary institutions through a formal HIV/AIDS curriculum and increased advocacy for HIV prevention and control.

Finally, our study also revealed that participants who were employed were three times more likely to test for HIV compared to their counterparts who were not working. In this study, more adolescents who were employed rated their distance to the health facility to be near compared to those unemployed. It is therefore likely that lack of transport fare hinders unemployed adolescents from reaching the health facility to seek care, thus low uptake of HIV testing. This result is similar to that of a scoping review study [26] where employment was found to be positively associated with HIV testing. This result highlights the need to target unemployed adolescents during HIV testing campaigns.

## 5. Limitations

This study had some limitations that must be considered in the interpretation and application of the results. This was a hospital-based study conducted in only three faith-based health facilities in Northern Uganda, so the findings of this study may not be representative of the whole of Uganda. Additionally, recall bias and social desirability bias were also possible limitations of this study since participants were required to recall and self-report information in response to the questions. However, recall bias was mitigated by narrowing the recall time to three months, while social desirability bias was controlled by the use of trained research assistants skilled in talking with adolescents.

## 6. Conclusion

Results of our study indicate that utilization of HIV testing services by adolescents aged 10-19 in Lira District, Northern Uganda, is generally low. Uptake of HIV testing services by adolescents is associated with completion of primary education, being employed, use of a condom in the previous sexual intercourse, and involvement in HIV testing outreaches. The findings of this study give insights into the utilization of HIV testing services and associated factors in Christian faith-based health institutions which are often surrounded by values of purity, abstinence, and fidelity. The results of the study can be used to inform the design of future strategies aimed at increasing uptake of HIV testing services in such institutions. More research is needed to understand and establish the causal relationships that exist between the utilization of HIV testing services and sociodemographic and individual factors.

## Figures and Tables

**Table 1:** Demographic and individual characteristics of adolescents aged 10-19 years in Lira District, Northern Uganda (*N* = 277), April 2021.

Characteristics	Frequency	Utilization of HTS		*p* value
	*n* (%)	No = 158	Yes = 119	
Age (years)				0.006^∗^
10-13	21 (7.6)	18 (11.4)	3 (2.5)	
14-17	121 (43.7)	74 (46.8)	47 (39.5)	
18-19	135 (48.7)	66 (41.8)	69 (58.0)	
Sex				0.831
Male	81 (29.2)	47 (29.7)	34 (28.6)	
Female	196 (70.8)	111 (70.3)	85 (71.4)	
Marital status				0.002^∗^
Single	163 (58.8)	105 (65.5)	58 (48.7)	
Married	79 (28.5)	30 (19)	49 (41.2)	
Cohabiting	24 (8.7)	14 (8.9)	10 (8.4)	
Divorced	7 (2.5)	6 (3.8)	1 (0.8)	
Widowed	4 (1.4)	3 (1.9)	1 (0.8)	
Level of education				0.006^∗^
No education	83 (30)	57 (86.1)	26 (21.8)	
Completed primary	92 (33.2)	53 (33.5)	39 (32.8)	
Completed “O” level	67 (24.2)	26 (16.5)	41 (24.2)	
Completed “A” level	11 (4.0)	8 (5.1)	3 (2.5)	
Tertiary education	24 (8.7)	14 (8.9)	10 (8.5)	
Employment status				≤0.001^∗^
Yes	120 (43.3)	54 (34.2)	66 (55.5)	
No	157 (56.7)	104 (8.9)	53 (44.5)	
Have a sexual partner				≤0.001^∗^
Yes	158 (57)	65 (41.1)	93 (78.2)	
No	119 (43)	93 (58.9)	26 (21.8)	
Age differences with partner(s) (*n* = 158)				0.232
0-1	26 (16.5)	7 (10.8)	19 (20.4)	
2-3	71 (44.9)	33 (50.8)	38 (40.9)	
>3	61 (38.6)	25 (38.5)	36 (38.7)	
Number of partners (*n* = 158)				0.557
0-1	139 (88.0)	56 (86.2)	83 (89.2)	
≥2	19 (12.0)	9 (13.8)	10 (10.8)	
Sexually active (*n* = 158)				0.023^∗^
Yes	144 (91.1)	55 (56.9)	89 (95.7)	
No	14 (8.9)	10 (15.4)	4 (4.3)	
Ever use of condom (*n* = 158)				≤0.001^∗^
Yes	67 (42.4)	14 (21.5)	53 (57.0)	
No	91 (57.6)	51 (78.5)	40 (43.0)	
Knowledge of partner's HIV status (*n* = 158)				≤0.001^∗^
Yes	118 (72.0)	37 (56.9)	81 (87.1)	
No	46 (28.0)	28 (43.1)	12 (12.9)	
Awareness of HIV testing & counseling services				0.997
Yes	226 (82.7)	107 (67.7)	119 (100)	
No	51 (18.4)	51 (32.3)	0 (0.0)	

HTS: HIV testing services. ^∗^Statistically significant variable at *p* < 0.05. “O”: ordinary secondary school level of education; “A”: advanced secondary school level of education.

**Table 2:** Facility-related factors affecting the utilization of HIV testing services among adolescents aged 10-19 years in Lira District, Northern Uganda (*N* = 277), April 2021.

Characteristics	Frequency	Utilization of HTS		*p* value
	*n* (%)	No = 158	Yes = 119	
Means to facility				0.379
Foot	148 (53.4)	79 (50.0)	69 (58.0)	
Bicycle/motorcycle	120 (43.3)	73 (46.2)	47 (39.5)	
Others	9 (3.2)	6 (3.8)	3 (2.5)	
Estimated distance to facility				0.263
<5 km	196 (70.8)	116 (73.4)	80 (67.2)	
>5 km	81 (29.2)	42 (26.6)	39 (32.8)	
Perceived distance to facility				0.763
Near	162 (58.5)	94 (59.5)	68 (57.1)	
Far	87 (31.4)	47 (29.7)	40 (33.6)	
Very far	28 (10.1)	12 (10.8)	11 (9.2)	
Facility opened by arrival time				0.030^∗^
Yes	264 (95.3)	146 (92.4)	118 (99.2)	
No	13 (47)	12 (7.6)	1 (0.8)	
Availability of service providers				0.001^∗^
Yes	219 (79.1)	113 (71.5)	106 (89.1)	
No	58 (20.9)	45 (28.5)	13 (10.9)	
Waiting time to receive care				0.940
0-1 hour	249 (89.0)	142 (89.9)	107 (89.9)	
2-3 hours	25 (9.0)	14 (8.9)	11 (9.2)	
>3 hours	3 (1.1)	2 (1.3)	1 (0.8)	
Adequacy of HTS				≤0.001^∗^
Yes	180 (65)	68 (43.0)	112 (94.1)	
No	97 (35)	90 (57.0)	7 (5.9)	
Involvement in HTS outreaches				≤0.001^∗^
Yes	76 (27.4)	16 (10.1)	60 (50.4)	
No	201 (72.6)	142 (89.9)	59 (49.6)	

^∗^Statistically significant variable at *p* < 0.05. HTS: HIV testing services.

**Table 3:** Multivariate logistic regression analysis of factors associated with HIV testing among adolescents aged 10-19 years in Lira District, Northern Uganda.

Variable	Utilization of HTS		cOR	95% CI	aOR	95% CI	*p* value
	No = 158	Yes = 119					
Level of education							
No education	57 (86.1)	26 (21.8)	0.64	(0.25-1.63)	2.75	(0.55-13.74)	0.217
Completed primary	53 (33.5)	39 (32.8)	1.03	(0.41-2.56)	5.47	(1.07-28.15)	0.042^∗^
Completed “O” level	26 (16.5)	41 (24.2)	2.21	(0.86-5.70)	11.16	(1.97-63.26)	0.060
Completed “A” level	8 (5.1)	3 (2.5)	0.53	(0.11-2.49)	0.56	(0.07-4.45)	0.562
Tertiary education	14 (8.9)	10 (8.5)	Ref		Ref	Ref	
Employment status							
Yes	54 (34.2)	66 (55.5)	2.4	(1.47-3.91)	2.77	(1.16-6.60)	0.022^∗^
No	104 (8.9)	53 (44.5)	Ref			Ref	
Use of condom (*n* = 158)							
Yes	14 (21.5)	53 (57.0)	4.83	(2.35-9.92)	4.46	(1.78-11.15)	0.001^∗^
No	51 (78.5)	40 (43.0)	Ref			Ref	
Involvement in HTS outreaches							
Yes	16 (10.1)	60 (50.4)	9.03	(4.81-16.94)	10.86	(3.81-30.93)	≤0.001^∗^
No	142 (89.9)	59 (49.6)	Ref			Ref	

Ref: reference category; cOR: crude odds ratio; aOR: adjusted odds ratio; HTS: HIV testing services; “O”: ordinary secondary school level of education; “A”: advanced secondary school level of education.

## Data Availability

The datasets used and/or analyzed during the study are available from the corresponding author on reasonable request.
